# Correlation Between Intracranial Carotid Artery Calcification and Prognosis of Acute Ischemic Stroke After Intravenous Thrombolysis

**DOI:** 10.3389/fneur.2022.740656

**Published:** 2022-04-13

**Authors:** Yuan Shen, Zhifeng Dong, Gang Xu, Jianguo Zhong, Pinglei Pan, Zhipeng Chen, Haicun Shi

**Affiliations:** ^1^Department of Neurology, Yancheng Third People's Hospital, Yancheng, China; ^2^Department of Neurology, The Yancheng School of Clinical Medicine of Nanjing Medical University, Yancheng, China; ^3^The Sixth Affiliated Hospital of Nantong University, Nantong, China; ^4^Department of Cardiology, Shanghai Jiao Tong University Affiliated Sixth People's Hospital, Shanghai, China; ^5^Department of Medical Imaging, Yancheng Third People's Hospital, Yancheng, China; ^6^Department of Central Laboratory, Yancheng Third People's Hospital, Yancheng, China

**Keywords:** acute stroke, calcification, prognosis, carotid artery, intravenous thrombolysis

## Abstract

**Objective:**

To investigate the correlation between prognosis and intracranial carotid artery calcification (ICAC) in patients with acute ischemic stroke (AIS) who receive intravenous thrombolysis (IVT).

**Methods:**

A total of 156 AIS patients who received IVT from March 2019 to March 2020 were enrolled. The modified Woodcock visual score was used to evaluate ICAC in nonenhanced head CT scans. Patients were divided into high calcification burden (HCB; score ≥3) and low calcification burden (LCB; score <3) groups. Demographic, laboratory, imaging and clinical data were compared between the two groups, and whether HCB was a prognostic factor was evaluated.

**Results:**

Compared with the LCB group, the HCB group had a higher incidence of atrial fibrillation (49.2 vs.22.1%, *P* < 0.001) and coronary heart disease (24.6 vs. 10.0%, *P* = 0.019) and higher serum homocysteine [15.31 (12.15, 17.50) vs. 14.40 (11.20, 16.20), *P* = 0.036] and hemoglobin A1c (6.93 ± 1.77 vs. 6.37 ± 0.74, *P* = 0.023) levels. Binary logistic regression analysis showed that atrial fibrillation (OR = 3.031, 95% CI: 1.312–7.006, *P* = 0.009) and HbA1c (OR = 1.488, 95% CI: 1.050–2.109, *P* = 0.026) were independent risk factors for ICAC. After adjusting for other risk factors, symptomatic-side and bilateral ICACs were independent risk factors for poor prognosis (OR = 1.969, 95% CI: 1.220–3.178, *P* = 0.006), (OR = 1.354, 95% CI: 1.065–1.722, *P* = 0.013) and mortality (OR = 4.245, 95% CI: 1.114–16.171, *P* = 0.034), (OR = 2.414, 95% CI = 1.152–5.060, *P* = 0.020) in patients with AIS who received IVT.

**Conclusion:**

ICAC is closely related to the prognosis of acute ischemic stroke after intravenous thrombolysis.

## Introduction

Thrombolysis is known to improve outcomes following acute ischemic stroke. Intravenous thrombolysis using recombinant tissue-type plasminogen activator (rt-PA) is still considered the first line of treatment (1). However, the effect of thrombolysis is influenced by many factors.

Intracranial carotid artery calcification (ICAC) was first observed with radiographic pathology in the early 1960s (2). Later studies found that 69.4% of Chinese patients (3) and 82.2% of Dutch patients who were >55 years had ICAC on conventional head computed tomography (CT) (4). Vascular calcification is part of the atherosclerotic process and may indicates severe stenosis (5). Atherosclerotic calcification occurs in the form of hydroxyapatite deposits that resemble bone mineralization (6). The more advanced stages consist of calcified plaques. Confirmation of the coronary calcium score as a predictor of future cardiac events has spurred researchers' interest in ICAC. Recent clinical studies have demonstrated the risk factors for intracranial arterial calcification and its clinical significance (7). Studies have shown that intracranial vascular calcification is related to a higher risk of stroke independent of cardiovascular risk factors (8, 9). However, in contrast to the coronary vasculature, few studies have investigated the relationship between ICACs and the prognosis of patients with acute ischemic stroke (AIS) who receive intravenous thrombolysis (IVT) (10–13). And the conclusions of these studies are controversial. The question of whether atherosclerotic calcification might have distinct prognostic effects on stroke patients and therefore warrant specific treatment strategies still needs to be investigated.

In this context, we examined the relationship of the burden of ICAC with poor outcome, complications and mortality in patients treated with IVT for AIS. Studying the effect of ICAC on the prognosis of IVT in AIS may not only help to understand the relationship between ICAC and stroke, but also modify the acute management strategy, because ICAC information is usually available before treatment decision-making (13).

## Methods

### Patients

Medical records of the patients registered in China Cerebrovascular Disease Registration Platform with discharged diagnosis of “AIS” between March 2019 to March 2020 in the stroke center of our hospital were reviewed.

### Inclusion Criteria

Inclusion criteria included the following: (1) 18 years of age or older; (2) diagnosis of AIS based on the diagnostic criteria of acute cerebral infarction formulated by the Compilation Committee of Chinese Cerebrovascular Disease Prevention Guidelines (14); (3) hospital admission within 4.5 h of ictus; and (4) receipt of standard thrombolytic treatment.

### Exclusion Criteria

In order to increase the safety, feasibility and rationality of the study and increase the homogeneity of the study object to a certain extent, the following cases are excluded: (1) posterior circulation infarction; (2) pre-stroke Modified Rankin Scale (mRS) score > 2; (3) standard contraindications for intravenous rt-PA therapy; (4) Patients receiving endovascular treatment; and (5) patients with malignant tumors, severe liver and kidney dysfunction or drug/alcohol abuse.

The study was approved by the ethics committee of our hospital.

### AIS Management

All AIS patients were treated with rt-PA at a dose of 0.9 mg/kg according to internationally recognized guidelines. Routine monitoring of neurological function scores and blood pressure was conducted. Cranial CT was repeated 24 h after thrombolysis to determine whether there was intracranial hemorrhage. If there was no intracranial hemorrhage, antithrombotic therapy and statin therapy were administered.

### Demographics and Detailed Clinical Data

The following information was collected from all patients: sex, age, and medical history, including hypertension, diabetes mellitus, hyperlipidemia, atrial fibrillation, coronary heart disease, and history of smoking or alcohol intake. Definition of hypertension: systolic blood pressure ≥ 140 mmHg or diastolic blood pressure ≥90 mmHg (measured at least three times at different times and in the same way at rest), or normal blood pressure when taking antihypertensive drugs; Definition of diabetes: dry mouth, polyuria, emaciation and other diabetes symptoms and random blood glucose ≥11.1 mmol/L, or fasting blood glucose ≥7.0 mmol/L, or oral glucose tolerance test (OGTT) 2 h blood glucose ≥11.1 mmol/L, (at least two times); Definition of dyslipidemia (serum triglycerides >1.7 mmol/L, low-density lipoprotein >3.4 mmol/L, total cholesterol >5.2 mmol/L or by the use of lipid-lowering agents); Atrial fibrillation and coronary heart disease were determined according to previous medical history and ECG, or was being treated for it. Smoking history: smokers who smoke more than 1 cigarette a day for more than 1 year; those who quit smoking for more than 15 years are equivalent to non-smoking; Definition of drinking: The conversion method of alcohol consumption and the standard of excessive drinking refer to the research of Kehui Liu et al. (15). Patient baseline characteristics were recorded: systolic and diastolic blood pressure, TOAST classification, onset to needle time (ONT) (min), door to needle time (DNT) (min), NIHSS while admitted, mRS score while admitted, and NIHSS on the seventh day. “Improvement” was defined as a decrease in the NIHSS score by ≥ 4 points at the 7th day after treatment. “Deterioration” was described as an increase of at least four points in NIHSS at the end of the 7th day. Complications of symptomatic intracranial hemorrhage (sICH) were registered. We investigated laboratory data on admission to our hospital, including data on glucose, total cholesterol, low-density lipoprotein, hemoglobin A1c (HbA1c), creatinine, C-reactive protein, homocysteine, and fibrinogen levels.

### Assessment of ICAC

**Figure 1:**
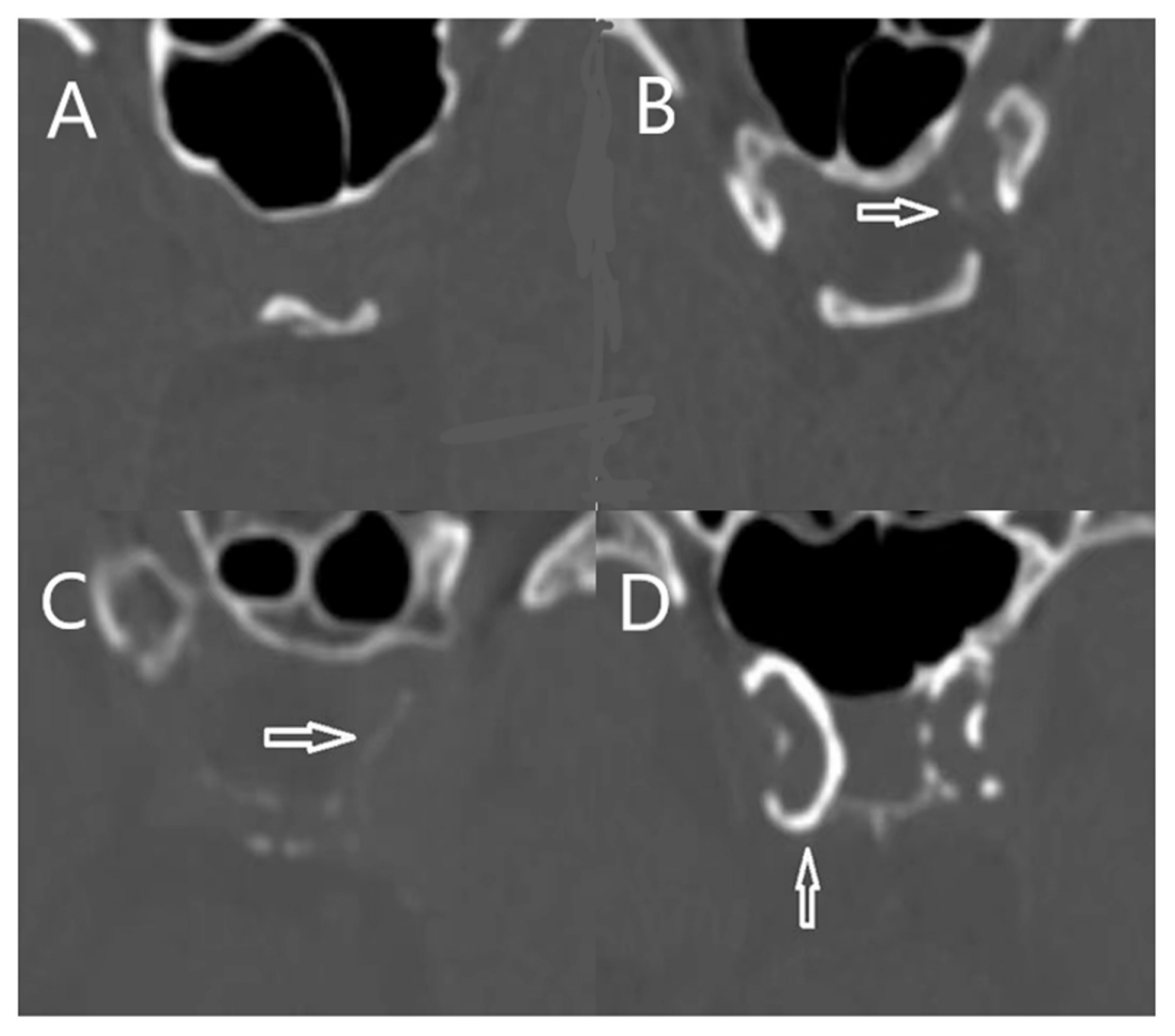
Intracranial internal carotid artery calcification score: **(A)**, Absence or near absence of calcifications, score = 1; **(B)**, thin and discontinuous calcification, score = 2; **(C)**, thick and discontinuous calcification, score = 3; **(D)**, thick and continuous calcification, score = 4.

All included patients underwent a non-contrast cranial CT axial scan (Discovery CT750HD CT Scanner; GE Healthcare, USA) at admission, all images were reconstructed at 0.625 mm axial sections. The ICAC scores were evaluated by a radiology specialist and a neurologist who were blinded to the clinical information, using the modified version of the Woodcock visual scoring (16). They had received standardized training before CT images evaluation. Bone window CT scans (modified window level: approximately 350 HU; width: 1,000 HU) through the skull base were used to calculate total carotid siphon calcification (TCSC) and symptomatic side calcification in the carotid siphon. The interrater agreement was excellent, the intraclass correlation coefficient from the two observers was 0.863 (*P* < 0.001). If the result was inconsistent, it should be decided by both parties through consultation. The calcification score of siphon segment represents the severity of internal carotid artery calcification. Absence or near absence of calcifications was recorded as one point; thin and discontinuous calcification was scored as two points; thin and continuous, or thick and discontinuous calcification was scored as three points; and thick and continuous calcification was recorded as four points (Figure 1). The calcification score was dichotomized into high calcification burden (HCB; score ≥3) and low calcification burden (LCB; score <3) groups (17, 18).

### Follow-Up

Patient neurological function was evaluated at 3 months after discharge using the mRS score by trained neurologists blinded to other clinical data. The patients who were unable to show up for the follow-up visits were subjected to the mRS score questionnaire via multiple phone calls.

The mRS score was dichotomized into favorable (mRS score 0–2 [good recovery, no significant disability, slight disability]) or unfavorable [mRS score 3–6 (moderate disability, moderately severe disability, severe disability, or death)].

### Statistical Analysis

SPSS for Windows, version 22.0 (IBM Corporation, New York, USA) was used for statistical analysis. Continuous variables were presented as the mean ± SD or median (interquartile range), and categorical variables were presented as percentages. The chi-square test was used for categorical data. The normally distributed variables were compared with independent *t*-tests, and the Mann-Whitney *U*-test was used for continuous non-normally distributed variables. Logistic regression was used for the multivariate analysis of baseline characteristics as risk factors for ICAC. The factors with statistical significance (*P* < 0.1) in univariate analysis were taken as independent variables and entered into logistic regression equation for statistical analysis. An enter method was used to design the final model. To further evaluate the interactive effect of atrial fibrillation and HbA1c, a new term (atrial fibrillation × HbA1c) was calculated and included in the relevant regression models as an additional variable. Multivariate logistic regression analyses were used to analyze whether ICAC was an independent risk factor for intracranial hemorrhage, poor prognosis at 3 months, and mortality after thrombolysis adjusted for potential influencing factors selected based on univariate analyses, *P* < 0.05 was taken as the cut-off for inclusion. *P* < 0.05 and 95% CIs of ORs (logistic regression) were considered statistically significant.

## Result

**Figure 2:**
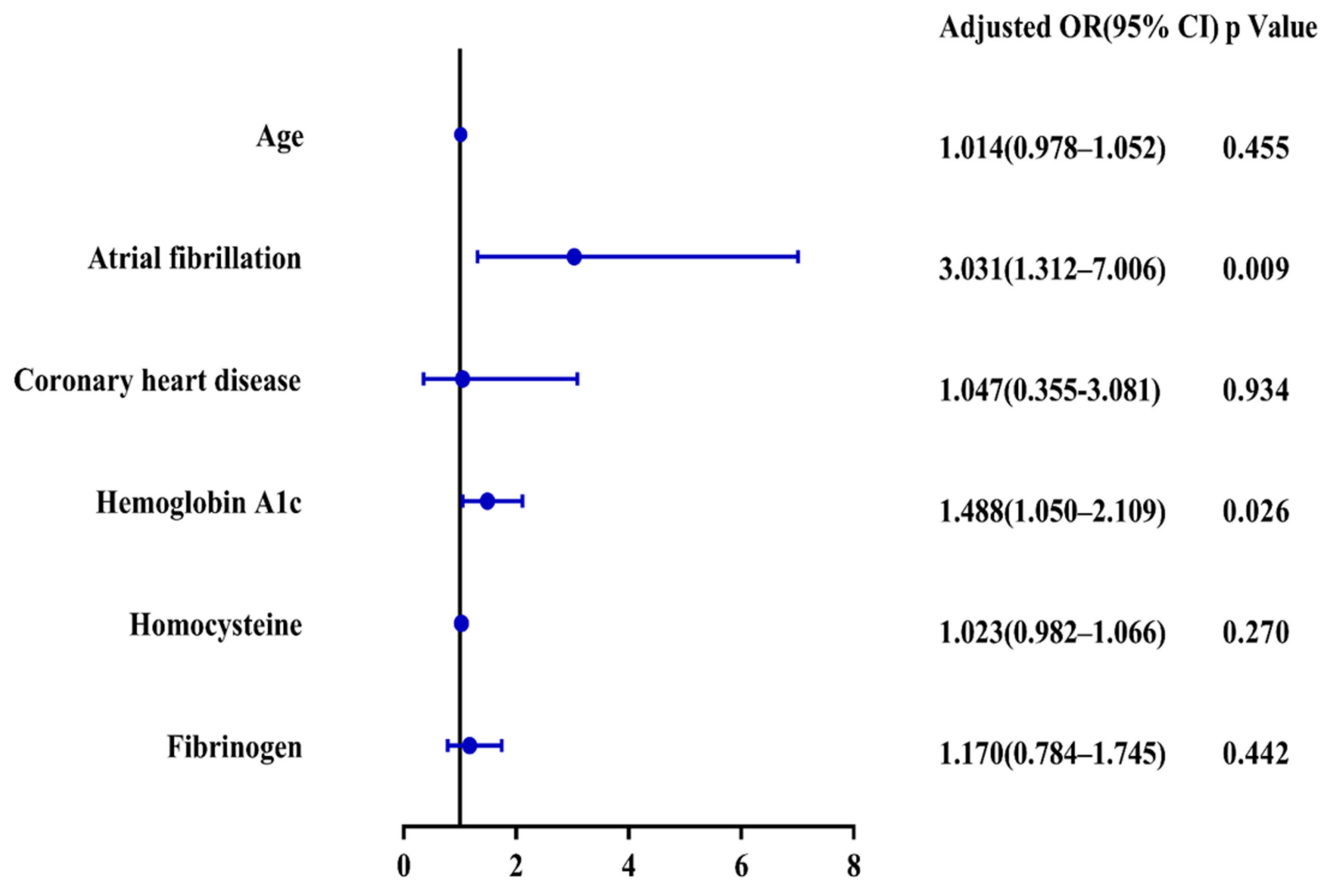
Risk factors for ICAC.

**Figure 3:**
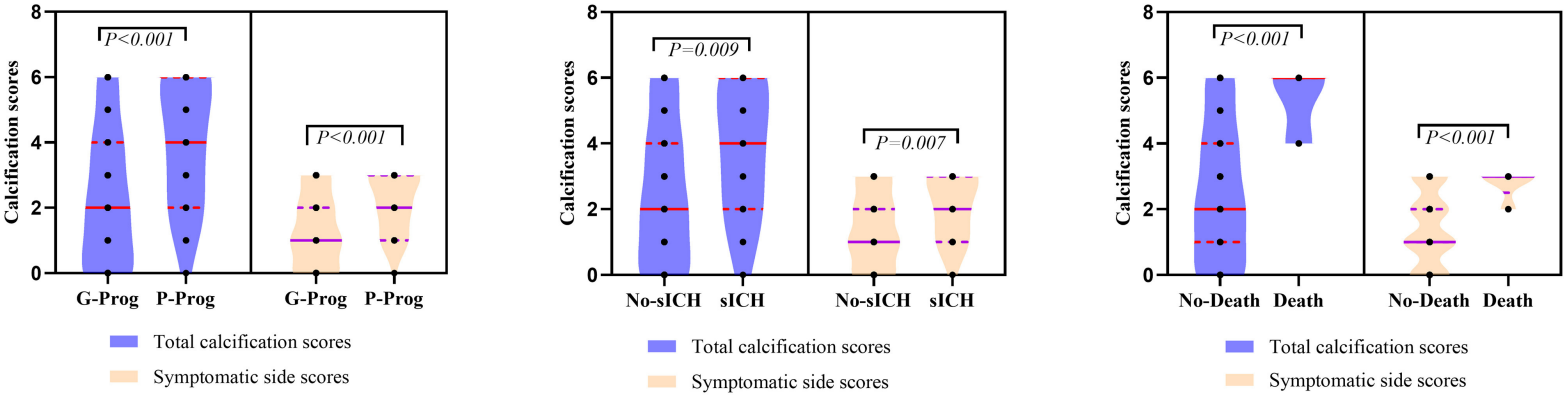
Comparation of symptomatic side and total modified Woodcock score of poor outcome, sICH and death.

A total of 156 patients were included in the study. A total of 61 patients (39.1%) had a high calcification burden (HCB). A comparison of the baseline characteristics of HCB patients and LCB patients was summarized in Table 1. Compared with patients in the LCB group, patients in the HCB group had a higher incidence of atrial fibrillation (49.2 vs. 22.1%, *P* < 0.001) and coronary heart disease (24.6 vs. 10.0%, *P* = 0.019), and higher HbA1c (6.93 ± 1.77 vs. 6.37 ± 0.74, *P* = 0.023) and higher serum homocysteine levels (15.31 [12.15, 17.50] vs. 14.40 [11.20, 16.20], *P* = 0.036). Stroke etiology, admission clinical parameters and clinical outcomes of HCB patients and LCB groups were compared. The NIHSS score while admitted to the HCB group was significantly higher than that of the LCB group [8.00 (3.00, 16.50) vs. 5.00 (2.00, 10.00), *P* = 0.004]. The NIHSS score on the 7th day was significantly higher in the HCB group than the LCB group [5.00 (0.50, 12.50) vs. 1.00 (0, 4.00), *P* < 0.001], and the proportion of deterioration (deterioration in NIHSS ≥4) in the HCB group was higher than that in the LCB group (11.5 vs. 2.1%, *P* = 0.029). The mRS score of the HCB group was higher than that of the LCB group at 3 months [3.00 (0, 4.50) vs. 1.00 (0, 3.00), *P* < 0.001] (Table 2). The potential risk factors for ICAC were listed in Figure 2. After correction for other risk factors, atrial fibrillation (OR = 3.031, 95% CI: 1.312–7.006, *P* = 0.009) and HbA1c (OR = 1.488, 95% CI: 1.050–2.109, *P* = 0.026) were independent risk factors for ICAC. The interaction term model showed that the interaction between atrial fibrillation and HbA1c was not significant (OR = 1.459, 95% CI: 0.503–4.440, *P* = 0.469). Clinical characteristics associated with poor outcome, sICH, mortality were shown in Table 3. Univariate analysis showed that symptomatic side and total modified Woodcock score were associated with poor outcome [1.00 (0, 2.00) vs. 2.00 (1.00, 3.00), *P* < 0.001], [2.00 (0, 4.00) vs. 4.00 (2.00, 6.00), *P* < 0.001], sICH [1.00 (0, 2.00) vs. 2.00 (1.00, 3.00), *P* = 0.007], [2.00 (0, 4.00) vs. 4.00 (2.00, 6.00), *P* = 0.009] and mortality [1.00 (1.00, 2.00) vs. 3.00 (2.50, 3.00), *P* < 0.001], [2.00 (1.00, 4.00) vs. 6.00 (4.00, 6.00), *P* < 0.001] (Figure 3). In the multivariate logistic regression analysis, symptomatic side and total modified Woodcock score were independently associated with poor outcome (OR = 1.969, 95% CI: 1.220–3.178, *P* = 0.006 and OR = 1.354, 95% CI: 1.065-1.722, *P* = 0.013) and death (OR = 4.245, 95% CI: 1.114–16.171, *P* = 0.034 and OR = 2.414, 95% CI: 1.152–5.060, *P* = 0.020), but not with sICH (OR = 1.200, 95% CI: 0.700–2.057, *P* = 0.508 and OR = 1.093, 95% CI: 0.833–1.435, *P* = 0.521), as shown in Table 4.

**Table 1:** Comparation of baseline characteristics of HCB and LCB groups.

**Group**	**Total (*n* = 156)**	**HCB (*n* = 61)**	**LCB (*n* = 95)**	***P***
Age (years)	70.81 ± 10.41	72.80 ± 11.91	69.54 ± 9.17	0.056
gende, male (*n*, %)	97 (62.2)	41 (67.2)	56 (58.9)	0.299
Hypertension (*n*, %)	110 (70.5)	44 (72.1)	66 (69.5)	0.722
Diabetes mellitus (*n*, %)	28 (17.9)	13 (21.3)	15 (15.8)	0.380
Hyperlipemia (*n*, %)	26 (16.7)	9 (14.8)	17 (17.9)	0.608
Coronary heart disease (*n*, %)	25 (16.0)	15 (24.6)	10 (10.0)	0.019
Atrial fibrillation (*n*, %)	51 (32.7)	30 (49.2)	21 (22.1)	<0.001
Smoking, (*n*, %)	36 (23.1)	11 (18.0)	25 (26.3)	0.231
Alcohol intake (*n*, %)	20 (12.8)	6 (9.8)	14 (14.7)	0.372
Systolic pressure (mmHg)	158.56 ± 24.54	158.85 ± 23.27	158.10 ± 26.61	0.854
Diastolic pressure (mmHg)	89.81 ± 13.02	89.65 ± 12.83	89.90 ± 13.22	0.906
Glycemia (mmol/L)	6.61 (5.43,7.87)	6.56 (5.32,8.30)	6.62 (5.44,7.71)	0.658
Total cholesterol (mmol/L)	4.14 ± 0.94	4.25 ± 0.99	4.08 ± 0.90	0.281
Low density lipoprotein (mmol/L)	2.39 ± 0.79	2.50 ± 0.87	2.32 ± 0.72	0.176
Lp-PLA2 (ng/ml)	446.17 ± 90.18	435.20 ± 140.97	451.62 ± 161.86	0.115
HbA1c (%)	6.59 ± 1.27	6.93 ± 1.77	6.37 ± 0.74	0.023
Homocysteine (umol/L)	14.88 (11.53,16.70)	15.31 (12.15, 17.50)	14.40 (11.20,16.20)	0.036
Creatinine (umol/L)	69.15 (59.00, 78.38)	71.23 (59.35 84.05)	64.50 (58.00,76.30)	0.136
CRP (mg/L)	3.82 (0.65, 6.41)	5.58 (0.61 7.52)	3.21 (0.65,6.22)	0.265
Fibrinogen (g/L)	2.94 (2.48, 3.32)	3.00 (2.74 3.73)	2.90 (2.37,3.25)	0.090

*HCB, high calcification burden; LCB, low calcification burden; Lp-PLA2, Lipoprotein-associated phospholipase A2; HbA1c, hemoglobin A1c; CRP, C-reactive protein*.

**Table 2:** Comparation of stroke etiology, admission clinical parameters and clinical outcomes of HCB and LCB groups.

**Group**	**Total (*n* = 156)**	**HCB (*n* = 61)**	**LCB (*n* = 95)**	***P*-value**
**TOAST classification**				0.015
Large artery atherosclerosis (*n*, %)	71 (45.5)	32 (52.5)	39 (41.1)	0.163
Lacunar infart (*n*, %)	57 (36.5)	14 (23.0)	43 (45.3)	0.005
Cardioembolism (*n*, %)	27 (17.3)	15 (24.6)	12 (12.6)	0.054
Undetermined inconplete evaluation (*n*, %)	1 (0.6)	0 (0)	1 (1.1)	1.000
ONT (min)	149.50 (116.25,192.75)	152.79 ± 53.35	152.24 ± 54.74	0.951
DNT (min)	50.24 ± 21.92	52.69 ± 25.20	48.67 ± 19.51	0.266
NIHSS while admitted	6.00 (2.00, 12.00)	8.00 (3.00, 16.50)	5.00 (2.00,10.00)	0.004
mRS while admitted	4.00 (2.00, 4.00)	4.00 (2.50, 5.00)	3.00 (2.00, 4.00)	0.028
NIHSS at seventh day	2.00 (0, 8.00)	5.00 (0.50, 12.50)	1.00 (0, 4.00)	<0.001
Improvement in NIHSS ≥4 (*n*, %)	51 (32.7)	19 (31.1)	32 (33.7)	0.742
Deterioration in NIHSS ≥4 (*n*, %)	9 (5.8)	7 (11.5)	2 (2.1)	0.029
Bleeding complication (*n*, %)	22 (14.1)	13 (21.3)	9 (9.5)	0.038
mRS at 3 months	1.00 (0,3.00)	3.00 (0,4.50)	1.00 (0, 3.00)	<0.001

*TOAST, Trial of Org 10172 in Acute Stroke Treatment; ONT, onset to needle time; DNT, door to needle time; NIHSS, National Institutes of Health Stroke Scale*.

**Table 3:** Univariate analysis: clinical characteristics associated with poor outcome, sICH, mortality.

	**Poor outcome**			**sICH**			**Mortality**		
	**Yes (*n* = 37)**	**No (*n* = 119)**	***P*-value**	**Yes (*n* = 22)**	**No (*n* = 134)**	***P*-value**	**Yes (*n* = 9)**	**No (*n* = 147)**	***P*-value**
Age (years)	76.41 ± 8.65	69.08 ± 10.33	<0.001	78.50 ± 7.58	69.55 ± 10.29	<0.001	79.78 ± 6.26	70.27 ± 10.38	0.007
sex, male (*n*, %)	17 (45.9)	80 (67.2)	0.020	10 (45.5)	87 (64.9)	0.081	5 (55.6)	92 (62.6)	0.730
Hypertension (*n*, %)	29 (78.4)	81 (68.1)	0.230	13 (59.1)	97 (72.4)	0.205	8 (88.9)	102 (69.4)	0.213
Diabetes mellitus (*n*, %)	7 (18.9)	21 (17.6)	0.860	2 (9.1)	26 (19.4)	0.370	3 (33.3)	25 (17.0)	0.204
Hyperlipemia (*n*, %)	5 (13.5)	21 (17.6)	0.556	2 (9.1)	24 (17.9)	0.536	1 (11.1)	25 (17.0)	1.000
Coronary heart disease (*n*,%)	11 (29.7)	14 (11.8)	0.009	10 (45.5)	15 (11.2)	<0.001	4 (44.4)	21 (14.3)	0.017
Atrial fibrillation (*n*, %)	21 (56.8)	30 (25.2)	<0.001	13 (59.1)	38 (28.4)	0.004	6 (66.7)	45 (30.6)	0.025
Smoking, (*n*, %)	3 (8.1)	33 (27.7)	0.013	0 (0)	36 (26.9)	0.006	0 (0)	36 (24.5)	0.091
Drinking histrory (*n*, %)	3 (8.1)	17 (14.3)	0.326	0 (0)	20 (14.9)	0.052	0 (0)	20 (13.6)	0.119
Systolic pressure (mmHg)	163.22 ± 31.09	16.88 ± 21.90	0.169	161.14 ± 25.49	157.93 ± 24.33	0.570	162.22 ± 23.43	158.15 ± 24.55	0.629
Diastolic pressure (mmHg)	91.03 ± 13.96	89.34 ± 12.67	0.492	90.68 ± 12.01	89.59 ± 13.15	0.715	94.11 ± 14.78	89.48 ± 12.85	0.299
Glycemia (mmol/L)	6.89 (6.10, 8.30)	6.47 (5.30, 7.71)	0.256	6.91 (5.61, 8.11)	6.59 (5.40, 7.84)	0.603	7.83 (6.62, 12.54)	6.50 (5.32, 7.71)	0.022
Total cholesterol (mmol/L)	4.16 ± 0.82	4.14 ± 0.97	0.922	4.05 ± 0.95	4.16 ± 0.94	0.620	4.51 ± 0.72	4.12 ± 0.95	0.228
Low density lipoprotein (mmol/L)	2.37 ± 0.75	2.40 ± 0.80	0.848	2.31 ± 0.76	2.40 ± 0.79	0.539	2.60 ± 0.62	2.38 ± 0.80	0.413
Lp-PLA2 (ng/ml)	446.09 (443.21, 468.85)	444.44 (441.47, 448.40)	0.098	446.22 (443.27, 449.59)	444.79 (441.68, 449.08)	0.293	451.65 (446.31, 615.27)	444.81 (441.75, 448.20)	0.007
HbA1c (%)	6.59 (6.25,6.64)	6.58 (6.20,6.62)	0.513	6.57 (6.25,6.61)	6.58 (6.20,6.63)	0.342	6.60 (6.53,7.00)	6.58 (6.20,6.63)	0.225
Homocysteine (umol/L)	15.31 (12.70,17.45)	14.70 (11.40,16.40)	0.180	16.07 (13.80,18.13)	14.65 (11.38,16.50)	0.041	16.80 (12.00,18.20)	14.88 (11.50,16.56)	0.401
Creatinine (umol/L)	70.80 (59.38, 79.95)	65.90 (58.00, 78.50)	0.402	72.75 (58.75, 86.00)	67.80 (58.75, 78.00)	0.203	72.10 (60.65, 89.70)	68.20 (58.00, 78.00)	0.386
CRP (mg/L)	6.11 (1.83, 7.65)	3.26 (0.50, 6.22)	0.072	2.11 (0.50, 6.53)	4.67 (0.66, 6.42)	0.389	7.36 (6.02,12.29)	3.21 (0.55,6.34)	0.012
Fibrinogen (g/L)	3.00 (2.50, 3.32)	2.93 (2.44, 3.37)	0.594	2.66 (2.36, 3.14)	2.96 (2.55, 3.37)	0.148	3.05 (2.86, 3.98)	2.93 (2.48, 3.31)	0.307
ONT (min)	150.00 (120.00,182.50)	146.00 (113.00,200.00)	0.758	145.50 (101.25,212.00)	150.00 (117.75,192.25)	0.931	157.00 (112.00,202.50)	147.00 (116.00,193.00)	0.595
DNT (min)	47.00 (31.50, 59.00)	47.00 (35.00, 59.00)	0.992	45.00 (30.25, 60.50)	47.00 (35.00, 58.25)	0.648	46.00 (36.00,84.50)	47.00 (34.00, 59.00)	0.487
NIHSS baseline	16.00 (11.00, 18.50)	4.00 (2.00, 8.00)	<0.001	13.00 (8.00, 17.00)	5.00 (2.00, 11.00)	<0.001	18.00 (17.00, 22.00)	5.00 (2.00, 11.00)	<0.001
sICH (*n*,%)	13 (35.1)	9 (7.6)	<0.001	—	—		2 (22.2)	20 (13.6)	0.615

**Table 4:** Univariate and multivariate regression models for the relationship between ICAC and poor outcome, sICH, and mortality.

	**Poor outcome**		**sICH**		**Mortality**	
**Modified woodcock scores**	**OR (95%CI)**	***P*-value**	**OR (95%CI)**	***P*-value**	**OR (95%CI)**	***P*-value**
**Crude association**						
Symptomatic side	2.210 (1.491–3.276)	<0.001	1.832 (1.161–2.891)	0.009	6.101 (1.641–22.680)	0.007
Contralateral side	1.893 (1.333–2.687)	<0.001	1.602 (1.065–2.412)	0.024	4.003 (1.612–9.941)	0.003
Total	1.471 (1.212–1.786)	<0.001	1.336 (1.068–1.670)	0.011	2.354 (1.332–4.159)	0.003
**Adjusted association**						
Symptomatic side	1.969 (1.220–3.178)	0.006	1.200 (0.700–2.057)	0.508	4.245 (1.114–16.171)	0.034
Contralateral side	1.341 (0.830–2.169)	0.231	1.154 (0.699–1.905)	0.576	3.514 (0.854–14.464)	0.082
Total	1.354 (1.065–1.722)	0.013	1.093 (0.833–1.435)	0.521	2.414 (1.152–5.060)	0.020

*Poor outcome, adjusted for gender, age, atrial fibrillation, coronary artery disease, smoking, NIHSS baseline, complication*.

*ICH, adjusted for age, atrial fibrillation, coronary artery disease, smoking, homocysteine, NIHSS baseline*.

*Mortality, adjusted for age, atrial fibrillation, coronary artery disease, glycemia, LpAa2, CRP, NIHSS baseline, TOAST classification, sICH, symptomatic intracranial hemorrhage*.

## Discussion

The retrospective patient cohort study including 156 acute ischemic stroke patients who received intravenous thrombolysis analyses risk factors for ICAC and the prognostic value of high ICAC for functional decline, intracranial hemorrhage and all-cause mortality during 3 months follow-up. The main findings of the study were that atrial fibrillation and HbA1c were independent risk factors for ICAC and that total ICAC score and ICAC score of the symptomatic side predicted the outcomes of functional decline, intracranial hemorrhage and all-cause mortality.

Although our study found that ICAC was closely related to atrial fibrillation, whether there was a causal relationship between ICAC and atrial fibrillation was uncertain. Atrial fibrillation may be a downstream reaction of ICAC. It is generally believed that most patients with ICAC have coronary artery calcification, which is a risk factor for atrial fibrillation (19–21).

Previous studies have found that diabetes mellitus or random blood glucose are independent risk factors for ICAC (11, 22, 23). Although our study did not find a correlation between immediate blood glucose and ICAC, we found that elevated glycosylated hemoglobin was an independent risk factor for ICAC. Glycosylated hemoglobin is a biomarker of average glucose levels that can be used to follow hyperglycemia over the long term (24). Levels of glycosylated hemoglobin are a biomarker for vascular health (25). The relationships between high HbA1c and ICAC might be explained by the negative effect of poor glycemic control. Artery calcification may be caused by the accumulation of advanced glycation end products (26). The important driving factors of atherosclerotic calcification in diabetes include endothelial dysfunction, oxidative stress, changes in mineral metabolism, increased production of inflammatory cytokines and release of osteoprogenitor cells from bone marrow to circulation (27).

ICAC is a manifestation of vascular aging and a risk factor for acute cerebrovascular disease. However, there are few studies on the effect of thrombolysis and the prognosis of acute ischemic stroke. Vascular calcification can be divided into two different forms in morphology, depending on the location, namely, in the intima or media (28). The effect of carotid artery calcification on vascular remodeling and intimal injury is still unclear (29). Some studies have shown that ICAC can lead to vascular stenosis, thus affecting the pass rate and success rate of intra-arterial mechanical thrombectomy (30).

The effect of ICAC on intravenous thrombolysis is still unclear. Xin et al. (10) found that after correcting other risk factors, ICAC had no effect on the efficacy and prognosis of intravenous thrombolysis. However, Wu et al.'s (31) study suggested that ICAC may form microemboli after intravenous thrombolysis to embolize distant small vessels, resulting in a poor thrombolytic effect or aggravation of symptoms. Our study found that patients with HCB in the siphon segment of the internal carotid artery had a worse response to thrombolysis than patients with LCB. The proportion of patients with HCB whose NIHSS score increased more than 4 points after thrombolysis was higher than that of patients with LCB. This result is consistent with the studies of Lee et al. and Tábuas-Pereira et al. (11, 22).

Considering that calcification is a sign of more severe atherosclerosis, we can reasonably expect that increased arterial calcification would impair autoregulation and make blood vessels more likely to rupture. However, whether ICAC is a risk factor for intracerebral hemorrhage after thrombolysis is still controversial. Lin et al. (17) asserted that ICAC was a risk factor for intracranial hemorrhage after thrombolysis. However, studies by Lee et al. and Tábuas-Pereira et al. (11, 22) suggested that there was no correlation between ICAC and symptomatic intracranial hemorrhage. In our study, after correcting for other confounding factors, ICAC was not an independent risk factor for post-thrombolytic hemorrhage. The reasons for these differences may include the following: (1). The research methods used in each study had different calcification scoring standards; (2). Selection bias and the coexistence of other covariates were not considered in other studies; (3). The research found that patients with HCB have a higher proportion of atrial fibrillation, and cardiogenic cerebral embolism caused by atrial fibrillation was prone to postinfarction hemorrhage, and therefore, the existence of these confounding factors led to the deviation of the results; and (4). Another contributor may be the type of calcification, which was not distinguished in any of these studies.

The cycle of repeated rupture and healing of the plaque, accompanied by inflammation of the core and fibrous cap, leads to the presence of heterogeneity and calcified carotid plaque (32). In animal models, vascular calcification is related to the severity of arteriosclerosis. Vascular calcification and decreased vascular elasticity can make organs more susceptible to the impact of high blood flow and systolic pressure fluctuations, resulting in decreased vascular perfusion (33–35). ICAC can reflect systemic vascular calcification. The higher mortality rate of patients with HCB after thrombolysis may be related to the higher rate of cardiovascular calcification, including aortic calcification. High coincident calcification may be associated with ischemic event recurrence, including stroke and acute coronary syndrome recurrence. In our study, there was significant difference in the prevalence of coronary heart disease between the two groups, and the effect on recurrent coronary heart disease after stroke was uncertain. In our study, after adjusting for other confounders, ICAC was an independent risk factor for mortality after thrombolysis in stroke. ICAC was associated with all-cause mortality in our cohort.

Lee et al. (22) found that the proportion of patients with HCB whose mRS score was higher than two points at 90 days was higher, while some other studies (10–12) posited that complicated with ICAC was not related to the three-month prognosis. After correcting for confounding factors such as sex, age, atrial fibrillation, coronary artery disease, smoking, NIHSS baseline, and complications, we found that ICAC was still an independent risk factor for poor prognosis of acute ischemic stroke at 3 months after thrombolysis. In our study, we found that HCB was positively related to the basic NIHSS score, which is an independent risk factor for poor prognosis (36–39). However, after correction of the basic NIHSS score, HCB was still an independent risk factor for poor prognosis at 3 months.

An important strength is that only few studies have investigated the relationship between ICAC and the prognosis of patients with acute ischemic stroke who receive intravenous thrombolysis. Yet, our study has several limitations. First, we investigated the prognosis of patients at only 3 months after discharge; longer-term follow-up studies are needed in the future. Second, this was a single-center study, and there was inevitably selection bias. And the sample size is relatively small, especially the sample of sICH and mortality, which leads to the imbalance of sample size. This could have affected the meaningfulness of results obtained. All of these limitations will be addressed in future studies by using a larger sample size. Third, patients who underwent revascularization treatment or received conventional conservative treatment within 4.5 h of onset were not included in the study. We would consider including such patients in the next study. Fourth, undoubtly, the un-calcified plaques were also identified as risk factor to their prognosis. However, in the original design of the project, only calcified plaques were studied, and the number and size of un-calcified plaques were not counted. The interaction between those morphologic characteristics of plaques will be added in future studies. And the types of calcification were not distinguished in our study.

## Conclusion

Our study showed that atrial fibrillation and glycosylated hemoglobin were independently associated with the prevalence of ICAC. ICAC was an independent risk factor for poor prognosis and mortality of cerebral infarction after intravenous thrombolysis. CT scans coupled with modeling algorithms will provide an objective marker for the prognosis of intravenous thrombolysis in acute cerebral infarction. Intervention of atrial fibrillation or control of high glycosylated hemoglobin may improve the prognosis of acute cerebral infarction after thrombolysis from another perspective.

## Data Availability Statement

The raw data supporting the conclusions of this article will be made available by the authors, without undue reservation.

## Ethics Statement

The studies involving human participants were reviewed and approved by Ethics Committee of the Yancheng Third People's Hospital. The patients/participants provided their written informed consent to participate in this study.

## Author Contributions

YS and ZD drafted the manuscript. YS, PP, and ZC participated in the literature collection. YS and GX analyzed the MRI features. HS and JZ reviewed and revised the manuscript. All authors read and approved the final manuscript.

## Funding

This work was supported by the Research Project Fund of Clinical College of Jiangsu Medical Vocational College (20209127).

## Conflict of Interest

The authors declare that the research was conducted in the absence of any commercial or financial relationships that could be construed as a potential conflict of interest.

## Publisher's Note

All claims expressed in this article are solely those of the authors and do not necessarily represent those of their affiliated organizations, or those of the publisher, the editors and the reviewers. Any product that may be evaluated in this article, or claim that may be made by its manufacturer, is not guaranteed or endorsed by the publisher.

## References

[1] Zhou Y Yan S Song X Gong Y Li W Wang M. Intravenous thrombolytic therapy for acute ischemic stroke in Hubei, China: a survey of thrombolysis rate and barriers. BMC Neurol. (2019) 19:202. 10.1186/s12883-019-1418-z 31438899 PMC6704516

[2] Ratinov G. Extradural intracranial portion of carotid artery, a clinicopathologic. Study. Arch Neurol. (1964) 10:66–73. 10.1001/archneur.1964.00460130070010 14089378

[3] Chen XY Lam WW Ng HK Fan YH Wong KS. The frequency and determinants of calcification in intracranial arteries in Chinese patients who underwent computed tomography examinations. Cerebrovas Dis. (2006) 21:91–7. 10.1159/000090206 16340183

[4] Bos D van der Rijk MJ Geeraedts TE Hofman A Krestin GP Witteman JC. Intracranial carotid artery atherosclerosis: prevalence and risk factors in the general population. Stroke. (2012) 43:1878–84. 10.1161/strokeaha.111.648667 22569939

[5] Chen XY Lam WW Ng HK Fan YH Wong KS. Intracranial artery calcification: a newly identified risk factor of ischemic stroke. J Neuroimag: Off J Am Soc Neuroimagg. (2007) 17:300–3. 10.1111/j.1552-6569.2007.00158.x 17894617

[6] Demer LL Tintut Y. Mineral exploration: search for the mechanism of vascular calcification and beyond: the 2003 Jeffrey M. Hoeg award lecture. Arterioscler Thromb Vasc Bio. (2003) 23:1739–739:1739–43. 10.1161/01.atv.0000093547.63630.0f 12958041

[7] Wu XH Chen XY Wang LJ Wong KS. Intracranial Artery Calcification and Its Clinical Significance. J Clin Neurol. (2016) 12:253–61. 10.3988/jcn.2016.12.3.253 27165425 PMC4960208

[8] Bugnicourt JM Leclercq C Chillon JM Diouf M Deramond H Canaple S. Presence of intracranial artery calcification is associated with mortality and vascular events in patients with ischemic stroke after hospital discharge: a cohort study. Stroke. (2011) 42:3447–447:3447–53. 10.1161/strokeaha.111.618652 21940971

[9] Bos D Portegies ML van der Lugt A Bos MJ Koudstaal PJ Hofman A. Intracranial carotid artery atherosclerosis and the risk of stroke in whites: the Rotterdam study. JAMA Neurol. (2014) 71:405–11. 10.1001/jamaneurol.2013.6223 24535643

[10] He XW Zhao R Li GF Zheng B Wu YL Shi YH. Lack of correlation between intracranial carotid artery modified woodcock calcification score and prognosis of patients with acute ischemic stroke after intravenous thrombolysis. Front Neurol. (2019) 10:696. 10.3389/fneur.2019.00696 31312173 PMC6614196

[11] Tábuas-Pereira M Sargento-Freitas J Silva F Parra J Mendes P Seara V. Intracranial carotid artery modified woodcock calcifi, et al. Intracranial internal carotid artery wall calcification in ischemic strokes treated with thrombolysis. Eur Neurol. (2018) 79:21–6. 10.1159/000477901 29131095

[12] Haussen DC Gaynor BG Johnson JN Peterson EC Elhammady MS Aziz-Sultan MA. Carotid siphon calcification impact on revascularization and outcome in stroke intervention. Clin Neurol Neurosurg. (2014) 120:73–7. 10.1016/j.clineuro.2014.02.021 24731580

[13] Gocmen R Arsava EM Oguz KK Topcuoglu MA. Atherosclerotic intracranial internal carotid artery calcification and intravenous thrombolytic therapy for acute ischemic stroke. Atherosclerosis. (2018) 270:89–94. 10.1016/j.atherosclerosis.2018.01.035 29407893

[14] Liu L Chen W Zhou H Duan W Li S Huo X. Chinese Stroke Association guidelines for clinical management of cerebrovascular disorders: executive summary and 2019 update of clinical management of ischaemic cerebrovascular diseases. Stroke Vasc Neurol. (2020) 5:159–76. 10.1136/svn-2020-000378 32561535 PMC7337371

[15] Liu K Ding Y Lu X Wang Z. Trends and socioeconomic factors in smoking and alcohol consumption among Chinese people: evidence from the 2008–2018 national health service surveys in Jiangsu Province. Arch Public Health. (2021) 79:127. 10.1186/s13690-021-00646-9 34243791 PMC8268563

[16] Subedi D Zishan US Chappell F Gregoriades ML Sudlow C Sellar R. Intracranial carotid calcification on cranial computed tomography: visual scoring methods, semiautomated scores, and volume measurements in patients with stroke. Stroke. (2015) 46:2504–504:2504–9. 10.1161/strokeaha.115.009716 26251250 PMC4542564

[17] Lin TC Chao TH Shieh Y Lee TH Chang YJ Lee JD. The impact of intracranial carotid artery calcification on the development of thrombolysis-induced intracerebral hemorrhage. J stroke Cerebrovasc Dis: off J National Stroke Ass. (2013) 22:e455–462. 10.1016/j.jstrokecerebrovasdis.2013.05.008 23800497

[18] Erbay S Han R Baccei S Krakov W Zou KH Bhadelia R. Intracranial carotid artery calcification on head CT and its association with ischemic changes on brain MRI in patients presenting with stroke-like symptoms: retrospective analysis. Neuroradiology. (2007) 49:27–33. 10.1007/s00234-006-0159-z 17089112

[19] Kirchhof P Bax J Blomstrom-Lundquist C Calkins H Camm AJ Cappato R. Early and comprehensive management of atrial fibrillation: executive summary of the proceedings from the 2nd AFNET-EHRA consensus conference 'research perspectives in AF'. Euro Heart J. (2009) 30:2969–77c. 10.1093/eurheartj/ehp235 19535417

[20] Schoonderwoerd BA Van Gelder IC Crijns HJ. Left ventricular ischemia due to coronary stenosis as an unexpected treatable cause of paroxysmal atrial fibrillation. J Cardiovasc Electrophysiol. (1999) 10:224–8. 10.1111/j.1540-8167.1999.tb00664.x 10090226

[21] Nishida K Qi XY Wakili R Comtois P Chartier D Harada M. Mechanisms of atrial tachyarrhythmias associated with coronary artery occlusion in a chronic canine model. Circulation. (2011) 123:137–46. 10.1161/circulationaha.110.972778 21200008

[22] Lee SJ Hong JM Lee M Huh K Choi JW Lee JS. Cerebral arterial calcification is an imaging prognostic marker for revascularization treatment of acute middle cerebral arterial occlusion. J stroke. (2015) 17:67–75. 10.5853/jos.2015.17.1.67 25692109 PMC4325637

[23] Yilmaz A Akpinar E Topcuoglu MA Arsava EM. Clinical and imaging features associated with intracranial internal carotid artery calcifications in patients with ischemic stroke. Neuroradiology. (2015) 57:501–6. 10.1007/s00234-015-1494-8 25633540

[24] Brantley JN Verla TD. Use of placental membranes for the treatment of chronic diabetic foot ulcers. Advances Wound Care. (2015) 4:545–59. 10.1089/wound.2015.0634 26339533 PMC4529081

[25] Willette AA Bendlin BB Colman RJ Kastman EK Field AS Alexander AL. Calorie restriction reduces the influence of glucoregulatory dysfunction on regional brain volume in aged rhesus monkeys. Diabetes. (2012) 61:1036–42. 10.2337/db11-1187 22415875 PMC3331743

[26] Lee HY Oh BH. Aging and arterial stiffness. Circ J: off J Japanese Circ Soc. (2010) 74:2257–22262. 10.1253/circj.cj-10-0910 20962429

[27] Yahagi K Kolodgie FD Lutter C Mori H Romero ME Finn AV. Pathology of human coronary and carotid artery atherosclerosis and vascular calcification in diabetes mellitus. Arterioscler Thromb Vasc Biol. (2017) 37:191–204. 10.1161/atvbaha.116.306256 27908890 PMC5269516

[28] Amann K. Media calcification and intima calcification are distinct entities in chronic kidney disease. Clin J Am Soc Nephrol: CJASN. (2008) 3:1599–605. 10.2215/cjn.02120508 18815240

[29] Laugesen E Høyem P Thrysoe S Hansen ESS Mikkelsen AFS Kerwin WS. Negative carotid artery remodeling in early type 2 diabetes mellitus and increased carotid plaque vulnerability in obesity as assessed by magnetic resonance imaging. J Am Heart Assoc. (2018) 7:e008677. 10.1161/jaha.118.008677 30369319 PMC6201412

[30] Hernández-Pérez M Bos D Dorado L Pellikaan K Vernooij MW López-Cancio E. A Intracranial carotid artery calcification relates to recanalization and clinical outcome after mechanical thrombectomy. Stroke. (2017) 48:342–7. 10.1161/strokeaha.116.015166 28008095

[31] Wu XH Chen XY Fan YH Leung TW Wong KS. High extent of intracranial carotid artery calcification is associated with downstream microemboli in stroke patients. J Stroke Cerebrovasc Dis: off J National Stroke Ass. (2017) 26:442–7. 10.1016/j.jstrokecerebrovasdis.2016.10.007 27818028

[32] Thompson T Shields KJ Barinas-Mitchell E Newman A Sutton-Tyrrell K. Calcified carotid artery plaques predict cardiovascular outcomes in the elderly. J Hyperten. (2015) 33:810–7. 10.1097/hjh.0000000000000488 25915886

[33] Niederhoffer N Lartaud-Idjouadiene I Giummelly P Duvivier C Peslin R Atkinson J. Calcification of medial elastic fibers and aortic elasticity. Hypertension. (1997) 29:999–1006. 10.1161/01.hyp.29.4.999 9095090

[34] Edmonds ME. Medial arterial calcification and diabetes mellitus. Z Kardiol. (2000) 89(Suppl 2):101–4. 10.1007/s003920070107 10769411

[35] O'Rourke MF Safar ME. Relationship between aortic stiffening and microvascular disease in brain and kidney: cause and logic of therapy. Hypertension. (2005) 46:200–4. 10.1161/01.hyp.0000168052.00426.65 15911742

[36] Adams HP Jr. Davis PH Leira EC Chang KC Bendixen BH Clarke WR. Baseline NIH stroke scale score strongly predicts outcome after stroke: a report of the trial of org 10,172 in acute stroke treatment (TOAST). Neurology. (1999) 53:126–31. 10.1212/wnl.53.1.126 10408548

[37] Almekhlafi MA Davalos A Bonafe A Chapot R Gralla J Pereira VM. Impact of age and baseline NIHSS scores on clinical outcomes in the mechanical thrombectomy using solitaire FR in acute ischemic stroke study. AJNR Am J Neuroradiol. (2014) 35:1337–40. 10.3174/ajnr.A3855 24557701 PMC7966577

[38] Li J Zhang P Wu S Yi X Wang C Liu M. Factors associated with favourable outcome in large hemispheric infarctions. BMC Neurol. (2018) 18:152. 10.1186/s12883-018-1148-7 30236075 PMC6149207

[39] Park H Kim BM Baek JH Kim JH Heo JH Kim DJ. Predictors of good outcomes in patients with failed endovascular thrombectomy. Korean J Radiol. (2020) 21:582–7. 10.3348/kjr.2019.0578 32323503 PMC7183835

